# Prevalence and factors that are associated with elevated blood pressure among primary school children in Mwanza Region, Tanzania

**DOI:** 10.11604/pamj.2020.37.283.21119

**Published:** 2020-11-30

**Authors:** Edson Elias Sungwa, Shangwe Ezekiel Kibona, Haruna Ismail Dika, Rose Mjawa Laisser, Helena Marco Gemuhay, Titus Kaizilege Kabalimu, Benson Richard Kidenya

**Affiliations:** 1 Department of Reproductive and Child Health, Hubert Kairuki Memorial University, Dar es Salaam, Tanzania,; 2 Department of Reproductive and Child Health, Ilemela Municipal Council, Mwanza, Tanzania,; 3 Department of Physiology, Catholic University of Health and Allied Sciences, Mwanza, Tanzania,; 4 Department of Maternal and Child Health, Catholic University of Health and Allied Sciences, Mwanza, Tanzania,; 5 Department of Paediatric Nursing, St. John's University of Tanzania, Dodoma, Tanzania,; 6 Department of Community Medicine, Hubert Kairuki Memorial University, Dar es Salaam, Tanzania,; 7 Department of Biochemistry, Catholic University of Health and Allied Sciences, Mwanza, Tanzania

**Keywords:** Prevalence, elevated blood pressure, overweight, obesity, Tanzania

## Abstract

**Introduction:**

hypertension (HTN) among children is reported to be increasing due to sedentary lifestyles. In developed countries the prevalence of paediatric HTN is recorded to be up to 21% while the magnitude of the same is up to 11% in Tanzania. This study aimed to determine the blood pressure profile and factors associated with elevated blood pressure (BP) among children of Mwanza region.

**Methods:**

a cross sectional study involving 742 children aged 6 to 16 years in selected primary schools in Mwanza region was conducted from June to August 2019. Data were collected using self-administered structured questionnaires where parents helped children to fill in. Blood pressure, body weight and height were measured using digital portable sphygmomanometer, self-calibrating digital weighing scale and Shorr measuring board respectively. Data were analyzed using EpiInfo.

**Results:**

this study found mean systolic blood pressure (SBP) and diastolic blood pressure (DBP) were 109.2 ± 8.1 mmHg and 62.3 ± 7.2 mmHg respectively. Prevalence of elevated BP was 18.1%. Pre-hypertension 9.6%, and hypertension 8.5%. The age specific elevated BP prevalence was significantly higher (OR = 1.9, 95% CI: 1.2 - 2.9, p = 0.008) among children aged ≥10 years (21.4%) than younger ones (15.1%). Prevalence was also higher (OR = 1.5, 95% CI: 1.1 - 2.3, p = 0.048) among girls (20.1%) than boys (16.0%). Elevated BP was found to be associated with obesity (OR = 3.5, 95% CI: 1.6 - 7.7, p = <0.001), overweight (OR = 1.9, 95% CI: 1.1 - 3.3, p = 0.037), eating fried food (OR = 2.2, 95% CI: 1.1 - 4.4, p = 0.023), drinking sugar soft drinks (OR = 2.0, 95% CI: 1.2 - 3.5, p = 0.002) and not eating fruits (OR = 13.4, 1.6, 95% CI: 2.1 - 65.8, p-value 0.006).

**Conclusion:**

findings indicate high prevalence of elevated BP among children of Mwanza region. There was an association between elevated BP and increased age, gender, sedentary lifestyle and obesity. Importance of measuring paediatric blood pressure and health information regarding effects of sedentary life is recommended to Tanzanians. Parents should encourage their children to have active physical activities. Moreover, health workers should implement programmes to modify sedentary lifestyle and prevent children from elevated blood pressure.

## Introduction

Paediatric hypertension (HTN) has increasingly become a concern among worldwide public health problems. Among non-communicable diseases, HTN is one of cardiovascular diseases which develop slowly and its pathogenesis often begins in childhood [1]. Blood pressure (BP) tracking from childhood to adulthood has shown that large number of adults worldwide suffering from essential HTN have had high BP since childhood. In addition, any child who develop high BP during childhood carries a great risk of developing HTN in adulthood [1,2]. It has been reported by several studies that all HTN outcomes in adulthood have their origin in childhood. Hence knowledge and skills on monitoring and controlling BP during childhood is vital [3]. Furthermore, cardiovascular and metabolic diseases in adulthood originate during childhood, therefore, checking BP is very important in assessing cardiovascular status of children. The increased trend in childhood HTN is attributed by a number of factors including obesity which occurs due to increased intake of diet low in vitamins, minerals and other healthy micronutrients. Similarly, intake of foods that are high in energy - dense fats and sugars lead to HTN [1,4]. Decreased physical activity levels due to the increasingly sedentary behavior such as motorized transportation, prolonged sitting also leads to HTN. In addition, seated screen time is among the factors that decreases energy expenditure by lowering basal metabolic rate and contribute to HTN [5]. Inhalation of cigarette smoke from active or passive smoker, also causes coronary vasoconstriction, coronary vascular resistance, and a decrease in coronary blood flow, hence, leads to HTN [6]. Paediatric HTN has familial tendencies, therefore, can be inherited from parents [7].

In their study on associated factors in high BP among school children in a middle size city, Southern Brazil, Costanzi *et al*. found 13.8% had HTN [8]. Another study that attempted to determine Variation in BP among adolescent school children in an urban slum of Kolkata, West Bengal reported the prevalence of paediatric HTN of 10.1% [9]. In their study Amritanshu *et al*. of India, reported that 4.7% of children and adolescents had hypertension, there was gradual increase over age and high among girls than boys [10]. In USA, as in other developed countries, the prevalence of paediatric HTN has been reported to be up to 21% [1]. Several studies have been conducted in Europe to determine the magnitude of paediatric HTN. In their studies Chiolero *et al*. of Switzerland and Maldonado *et al*. of Portugal reported the prevalence of paediatric HTN of 2.2% and 12.8% respectively [11,12]. In Africa HTN among children and adolescent, which was rare in the past, is now rapidly increasing. A study conducted in Ghana reported that BP increases with increasing age in rural, but also semi - urban and urban areas. It was further reported that, rural girls had higher systolic and diastolic BP than semi urban boys [13]. A review conducted by Noubiap *et al*. on 76 published studies found the prevalence of paediatric HTN was 5.5% [14]. Studies conducted in Nigeria by Ujunwa *et al*. and Also *et al*. reported prevalence of elevated BP among children to be 22.7% and 3% respectively [15,16]. Concomitant with high prevalence of HTN in developed countries, the magnitude in Tanzania is increasing as well. In their studies, Chillo *et al*. Mushengezi *et al*. and Muhihi *et al*. conducted in Dar es Salaam showed paediatrics hypertensive were 3.9%, 4% and 11% respectively [17-19].

In North West Tanzania; studies have shown a high population prevalence of hypertension among adults ranging from 8% to 16.4% [20-22]. The magnitude of adult hypertension is known, yet little is known about paediatric hypertension hence the importance of this current study. The current reports on the increase of non-communicable diseases, have also reported renal dysfunction among children in northwestern Tanzania at 16.2% [23]. The prevalence of renal dysfunction suggests a hidden burden of elevated BP among children. BP is not routinely checked in children and there is limited information about its profile particularly in Mwanza and other parts of Tanzania. Therefore this study aimed to determine the magnitude of the problem in the study setting, providing BP profile, the factors associated with elevated BP among primary school children in Mwanza region.

## Methods

**The design, setting and participants of the study:** a cross sectional study was carried out for a period of three months among primary school children aged 6-16 years, from 7 randomly selected primary schools in North West Tanzania, Mwanza city. A minimum sample size of 742 primary school children recruited to participate in this study. The city of Mwanza is located on the southern shores of the second largest freshwater lake in the world, the Lake Victoria. It is also considered the second largest city in Tanzania.

**Data collection procedure:** children, whose parents or guardians agreed to consent and allow their children to participate in the study, were enrolled. Information regarding gender, age, tribes, family history of hypertension, means of transport to school, type of food eaten, time spent on watching TV at home, time spent video gaming, participation in house chores, and participation in sports and games were collected using pretested structured questionnaire. Body weight was measured using a self - calibrating precision digital scale (Omron, Japan). Height was measured to the nearest 0.1 cm by a fixed Shorr measuring board (Shorr Productions). Children were asked to take off their shoes and any over clothing for the height and weight measurements. They were asked to stand on the weighing scale and measurements were rounded to one decimal place. Blood pressure was measured using digital, portable automatic BP monitor with the proper cuff size for children of 9 x 18 cm (CONTEC 08A, Hamburg, Germany). CONTEC blood pressure machine is small but easy to read display. It has good useful up to date features; is easy to use, and the most important issue, gives reliable readings. The cuff bladder that usually covers 80%-100% of the circumference of the arm was appropriate [24,25]. Delay for at least 5 to 10 minutes before taking BP was allowed until child was relatively calm. Blood pressure was measured while the child was sitting with his/her arm at heart level with the back supported and feet uncrossed on the floor [24,25]. Three separate readings of BP at least 5-10 minutes apart were measured and averaged later [1]. A child who was found to have elevated BP, his/her BP was measured 3 times at an interval of at least 6 hours to justify criteria of hypertension. Either persistently average SBP and/or average DBP ≥95^th^ percentile was considered as hypertension [19,25]. Blood pressure status was classified according to SBP and/or DBP percentiles [26-28] as follows: i) Normal blood pressure: average SBP and/or average DBP <90^th^ percentile. ii) Pre-hypertension: average SBP and/or average DBP ≥90^th^ percentile but <95^th^ percentile. iii) Hypertension: average SBP and/or average DBP ≥95^th^ percentile. To increase the validity and reliability of the data collection, the research instruments were pretested. On a scheduled day, the principle investigator and the researcher assistants, altogether went to a different school to collect data. Two trained researcher assistants conducted anthropometric measurements in a special prepared room at each school early in the morning before classes. Principle investigator conducted BP measurements and supervised researcher assistants who were measuring weight and height respectively. BP measurements were done following best BP measurement practices [25], the anthropometric measurements were carried out in accordance with the WHO recommendations [29].

**Data analysis:** data were entered into a computer using Excel 2013, cleaned and analyzed using Epi Info version 7.2.2.6. Continuous variables were reported using means and standard deviations. Categorical data were reported as whole numbers and percentages. Descriptive statistics (means, standard deviations and frequencies) were used to describe the general characteristics of the participants. Chi-square test (χ^2^) test and logistic regression were used to determine association between elevated BP and various categorical variables. One-way ANOVA was used to determine associated between age group with mean SBP and DBP. The 95% confidence interval was determined and predictors with p - value of less than 0.05 was considered statistically significant.

**Ethical considerations:** ethical clearance for this study was sought from the joint Catholic University of Health and Allied Sciences (CUHAS)/ Bugando Medical Centre (BMC) research ethics and review committee (CREC) with ethical clearance number CREC/374/2019. Permission to conduct the study in the selected schools was sought from the respective district administrative secretaries and from head teachers of selected schools. Informed consent/assent to participate in the study were sought from the parents/guardians and children before enrollment to the study.

## Results

**Socio-demographic characteristics and anthropometric measurements of study participants:** a total of 742 children (males = 368, females = 374) from seven primary schools in Nyamagana and Ilemela districts were enrolled in this study (Table 1). The participants aged 6 - 16 years, with a mean age of 10.8 ± 2.4 years. More than 50% of participants were from urban areas and majority of them were from public schools (Table 1). Mean height of the children who participated in this study was 139.3 ± 12.8 cm and body mass index (BMI) was 16.6 ± 2.4. Majority 589 (79.4%) of them had normal BMI (Table 1). The minimum and maximum SBP obtained from children who participated in this study were 86 mmHg - 138 mmHg and the DBP were 42 mmHg - 98 mmHg. Both SBP and DBP were not normally distributed. The median SBP and DBP were 109 mmHg (98 - 120 mmHg IQR) and 62 mmHg (52 - 71 mmHg IQR) respectively. Less than 50%of children who participated in this study reported that they were given pocket money of various amounts to spend to school (Table 1). Majority of school children who participated in this study (58.5%), reported going to and from school mostly on feet, while others used school bus, private cars, or public transport (Table 1).

**Table 1:** socio-demographic and anthropometric characteristics of primary school children at Mwanza, Tanzania (N=742)

Characteristics	Mean ± SD,	IQR, Median (N)	(%)
**Age**	10.8 ± 2.4		
**Age category**	≤ 10	392	(52.8)
	> 10	350	(47.2)
**Gender**	Female	374	(50.4)
	Male	368	(49.8)
**Type of schools**	Private	272	(36.7)
	Public	470	(63.3)
**Place of residence**	Rural	350	(47.2)
	Urban	392	(52.8)
**Height**	139.3 ± 12.8		
**Normal BMI (5^th^ - 85**^th^ percentile)	13.5 - 19.0		
**BMI (mean)**	16.6 ±2.4		
**BMI category**	Underweight	40	(5.4)
	Normal	589	(79.4)
	Overweight	75	(10.1)
	Obesity	38	(5.1)
**SBP mmHg (mean)**	109.2 ± 8.1		
**DBP mmHg (mean)**	62.3 ±7.2		
**SBP (mmHg)** (> 90 percentile (IQR))		98 - 120 (109)	
**DBP (mmHg)** (> 90 percentile (IQR))		52 - 71 (62)	
**Blood pressure**	Normal BP	608	(81.9)
	Pre - hypertension	71	(9.6)
	Hypertension	63	(8.5)
**Elevated SBP**	Yes	69	(9.3)
	No	673	(90.7)
**Elevated DBP**	Yes	647	(87.2)
	No	95	(12.2)
**Elevated SBP + DBP (combined)**	Yes	134	(18.1)
	No	608	(81.9)
**Pocket money**	≤ 500 Tanzanian shillings	327	(44.0)
	> 500 Tanzanian shillings	28	(3.8)
	Not given	387	(52.2)
**Transport to school**	Walking	434	(58.5)
	Not walking	308	(41.5)
**Name of schools**	Alliance Medium primary school	106	(14.3)
	Bugogwa primary school	108	(14.6)
	Furaha English Medium primary school	104	(14.0)
	Green view Medium primary school	100	(13.5)
	Ilemela primary school	102	(13.7)
	Lwanhima primary school	108	(14.6)
	Pamba primary school	114	(15.3)

**Mean systolic and diastolic blood pressure among boys and girls of different age groups in Mwanza region:** mean SBP and DBP of study participants were 109.2 ± 8.1 mmHg and 62.3 ± 7.2 mmHg respectively. Distribution of the mean BP for boys and girls of participants in this study is shown in Table 2. There are gradual increases of SBP by a single unit or two (mmHg) as age increases. Female children have significantly higher SBP than male children in all ages except for age 8, 11 and 16 where males have higher SBP than females. No significant difference in DBP by increases in age, however; is noted that females have higher DBP than males except at age 8 where both gender have the same value while age 12, 13 and 16 males have higher DBP than females.

**Table 2:** distribution of blood pressure for boys and girls at different age groups among primary school at Mwanza, Tanzania (N=742)

Age (Years)	Blood pressure (mmHg)			
	Boys		Girls	
	Systolic	Diastolic	Systolic	Diastolic
6	103	61	112	64
7	104	61	107	66
8	110	63	107	63
9	109	63	112	68
10	111	65	114	69
11	113	66	111	64
12	109	66	116	64
13	113	65	114	61
14	109	63	117	64
15	113	63	118	73
16	116	67	114	66

**Figure 1:**
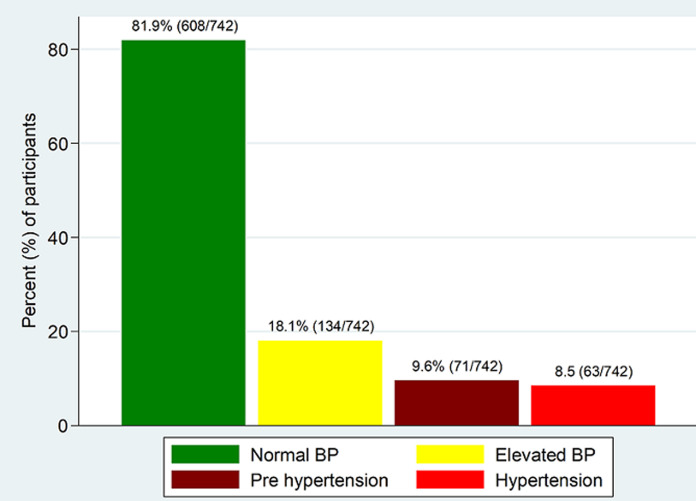
prevalence of elevated blood pressure (BP) and hypertension among primary school at Mwanza, Tanzania (N=742)

**Prevalence of elevated blood pressure and hypertension:** using western adopted reference values (normal: BP<120/80 mmHg; prehypertension: BP 120/80 mmHg - 130/80 mmHg and hypertension: BP ≥131/80 mmHg) [25], the prevalence of elevated BP was 18.3%, pre-hypertension and hypertension being 10.5% and 7.8% respectively. However, using population specific reference ranges derived from this study, the prevalence of elevated BP is 134 (18.1%) (pre-hypertension - 9.6% and hypertension - 8.5%) as shown in Figure 1.

**Figure 2:**
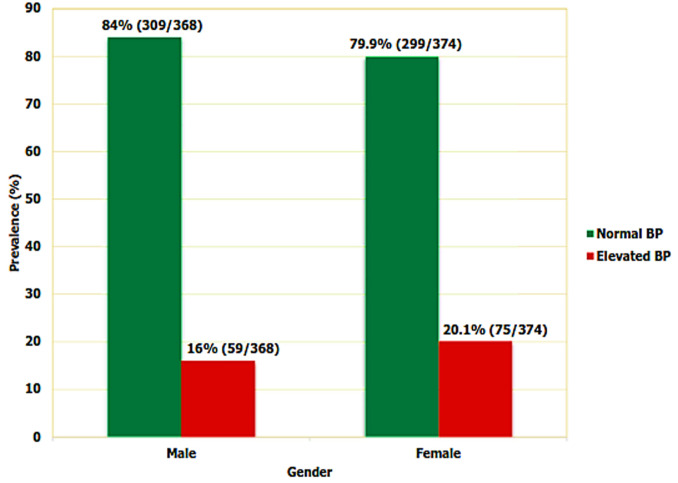
elevated blood pressure by gender among primary school at Mwanza, Tanzania (N=742)

**Figure 3:**
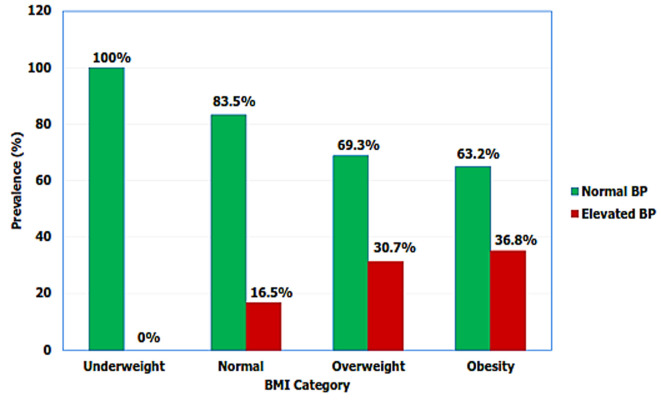
elevated blood pressure by body mass index among primary school at Mwanza, Tanzania (N=742)

**Factors associated with elevated blood pressure among primary school children in Mwanza region:** several factors were significantly associated with increased odds for elevated BP in univariate regression analysis (Table 3). These included age 10 years and above, obesity, overweight, eating fried food and drinking light sugar soft drinks. However, children who reported to be living in urban were less likely to have elevated BP than children who reported to be living in rural areas (OR = 0.6, 95% CI: 0.4 - 0.8, p - value 0.008) as shown in Table 3. Moreover, gender difference was not found to be associated (OR = 1.3, 95% CI: 0.9 - 1.9, p - value 0.154) with elevated BP (Figure 2). In multivariate analysis after adjusting for all significant variables (p - value < 0.05) from the univariate regression analyses; age, gender, studying in private schools, overweight, obesity, eating fried food, drinking sugar soft drinks and not eating fruits were found to be associated with elevated BP among children. Older children 10 years and above had higher odds for elevated BP (OR = 1.9, 95% CI: 1.2 - 2.9, p - value 0.008) than below 10 years children. Although no association was observed in univariate analysis female children had 1.5 odds higher for having elevated BP compared to male children of the same age (OR = 1.5, 95% CI: 1.1 - 2.3, p - value 0.048) in multivariate analysis. Furthermore, attending private schools is associated with elevated BP. Children who reported to attend private schools had 3.5 times higher odds (OR = 3.5, 95% CI: 1.4 - 9.0, p - value <0.001) for elevated BP than children studying in public schools. Overweight and obese children had almost two to four times higher odds for elevated BP compared to the ones with normal weight (OR = 1.9, 95% CI: 1.1 - 3.3, p - value 0.037) and (OR = 3.5, 95% CI: 1.6 - 7.7, p value <0.001) respectively. Furthermore, as BMI increases BP increases as well (Figure 3). Children who reported to be given money to buy food and drinks at school were more likely to have elevated BP than those not usually given money (OR = 1.7, 95% CI: 1.1 - 3.0, p - value 0.034). Compared to children who reported to not eating fried food or not drinking sugar soft drinks; children who reported being eating fried food or drinking sugar soft drinks had 2 times the odds for elevated BP (OR = 2.2, 95% CI: 1.1 - 4.4, p - value 0.023) and (OR = 2.0, 95% CI: 1.2 - 3.5, p - value 0.012) respectively. Again, not eating fruits is significantly associated with elevated BP (OR = 13.4, 1.6, 95% CI: 2.1 - 65.8, p - value 0.006).

**Table 3:** univariate and multivariate logistic regression analysis on factors associated with elevated BP among primary school children (N=742)

Variables	Blood pressure		Crude OR		Adjusted OR	
	Elevated	Normal	OR (95% CI)	P-value	OR (95% CI)	P-value
Age category						
≤ 10	59 (44.0)	333 (54.8)	1.0		1.0	
> 10	75 (56.0)	275 (45.2)	1.5 (1.1 - 2.2)	**0.024**	1.9 (1.2 - 2.9)	**0.008**
**Gender**						
Male	59 (16.0)	309 (84.0)	1.0		10.	
Female	75 (20.0)	299 (80.0)	1.3 (0.9 - 1.9)	0.154	1.5 (1.1 - 2.3)	**0.048**
**Residence**						
Rural	77 (57.5)	273 (44.9)	1.0			
Urban	57 (42.5)	335 (55.1)	0.6 (0.4 - 0.8)	**0.008**	0.3 (0.1 - 1.0)	< 0.001
**Type of schools**						
Public schools	89 (66.4)	381 (62.7)	1.0			
Private schools	45 (33.6)	227 (37.3)	0.8 (0.5 - 1.2)	0.412	3.5 (1.4 - 9.0)	**< 0.001**
**BMI category**						
Normal	97 (72.4)	492 (80.9)	1.0		1.0	
Underweight	0 (0.0)	40 (6.6)	0.0 (0.0 - 0.0)	0.969		
Overweight	23 (17.2)	52 (8.6)	2.2 (1.3 - 3.8)	**0.003**	1.9 (1.1 - 3.3)	**0.037**
Obesity	14 (10.5)	24 (4.0)	2.9 (1.4 - 5.9)	**0.002**	3.5 (1.6 - 7.7)	**< 0.001**
**Pocket money given**						
No	61 (45.5)	329 (51.1)	1.0		1.0	
Yes	73 (54.5)	279 (45.9)	1.4 (0.9 - 2.0)	0.071	1.7 (1.1 - 3.0)	**0.034**
**Watching television**						
No	42 (31.3)	235 (38.6)	1.0		1.0	
Yes	92 (68.7)	373 (61.4)	1.4 (0.9 - 2.1)	0.109	0.9 (0.5 - 1.5)	0.693
**Playing video/computer games**						
No	128 (95.5)	596 (98.0)	1.0			1.0
Yes	6 (4.5)	12 (2.0)	2.3 (0.9 - 6.3)	0.115	1.3 (0.8 - 2.3)	0.325
**Eating fried food**						
No	13 (9.7)	121 (19.9)	1.0		1.0	
Yes	121 (90.3)	487 (80.1)	2.3 (1.2 - 4.2)	**0.003**	2.2 (1.1 - 4.4)	**0.023**
**Drinking sugar soft drinks**						
No	25 (18.7)	192 (31.6)	1.0			
Yes	109 (81.3)	416 (68.4)	2.0 (1.3 - 3.2)	**0.002**	2.0 (1.2 - 3.5)	**0.012**
**Drinking fruit juices**						
Yes	122 (91.0)	576 (94.7)	1.0		1.0	
No	12 (9.0)	32 (5.3)	1.8 (0.9 - 3.5)	0.119	0.7 (0.1 - 3.6)	0.707
**Eating fruits**						
Yes	124 (92.5)	599 (98.5)	1.0		1.0	
No	10 (7.5)	9 (1.5)	5.3 (2.1 - 13.4)	**0.001**	13.4 (2.1 - 65.8)	**0.006**
**Eating vegetables**						
Yes	121 (90.3)	570 (93.8)	1.0		1.0	
No	13 (9.7)	38 (6.2)	1.6 (0.8 - 3.1)	0.170	1.6 (0.8 - 3.2)	0.149
**Positive family history of HTN**						
No	124 (92.5)	566 (93.1)	1.0		1.0	
Yes	10 (7.5)	42 (6.9)	1.1 (0.5 - 2.2)	0.821	1.1 (0.6 - 2.3	0.729
**Exposure to passive smoking**						
No	119 (88.8)	539 (88.7)	1.0		1.0	
Yes	15 (11.2)	69 (11.3)	1.0 (0.5 - 1.7)	0.959	1.0 (0.7 - 1.9	0.958

## Discussion

**Prevalence of elevated blood pressure:** this study was able to determine the BP profile and factors associated with elevated BP among primary school children at Mwanza region. Mean SBP and DBP of study participants were 109.2 ± 8.1 mmHg and 62.3 ± 7.2 mmHg respectively. The median SBP and DBP were 109 mmHg (98 - 120 mmHg IQR) mmHg and 62 mmHg (52 - 71 mmHg IQR) respectively. The study found that a significant proportion of primary school children had elevated BP. The prevalence of elevated BP using western reference value and population specific reference is 18%. The prevalence for hypertension was 7.8% when using western reference values and 8.5% when using population specific reference ranges derived from this study. Study shows that by using western reference values we miss some children with hypertension than when using population specific reference ranges derived from this study. These findings agree with findings reported by Lackland *et al*. that African Americans have consistently higher BP than Caucasians counterparts [30,31]. The risks for elevated BP increases in children who use less active mode of transport to and from school i.e. children who are studying in private schools. Obesity and overweight, eating fried food, drinking sugar soft drinks, female children, increased age and having money to spend to school were other factors associated with elevated BP.

Previous studies in Tanzania [17-19] and elsewhere [15,16] reported low prevalence of elevated BP than in this study, while others have reported higher prevalence [15,16,19] respectively. Moreover, prevalence of HTN in this study is lower than what was reported by Chilo *et al*. Mushengezi *et al*. and Muhihi *et al*. [17-19] respectively, but higher than in Nigeria by Ujunwa *et al*. [15]. The prevalence of HTN in this study is higher than in other developed countries [3,10,11]. There are several plausible reasons for such a high rate of elevated BPs. Participants in this study have never been measured their BP before. Despite of the fact that, assurance was made before BP measurement and clear explanation of the procedure was given to study participants, but when cuff squeezes their arms to measure BP some participants had cuff filling induced anxiety and fear. They also had stranger anxiety (agitated, avoidance, clenching teeth, fist clenching). Moreover, when BP machine pumped up to measure their BP it triggered some pain and made some of them uncomfortable. These effects triggered a sympathetic nervous system response such as tachycardia and elevated BP through a neurohormonal cascade. Poor *et al*. found similar pattern on benefit of BP measurement in paediatrics [32]. Other countries such as United States [1], Portugal [12], Southern Brazil [8] and West Bengal [9] have reported higher prevalence than this possibly because of life style and ethnicity.

**Factors associated with elevated blood pressure:** study found that age is associated with elevated BP, older children had almost twice odds to have elevated BP than young children. These findings of higher prevalence of elevated blood BP in older children concur with other studies conducted elsewhere [19]. Age related increase in blood pressure is attributable to increasing weight with age and changes in arterial and arteriolar stiffness. There is correlation between BP and BMI in children which highlights the development of metabolic syndrome [10,15]. Findings showed that girls have higher BP than boys. Ujunwa *et al*. found similar pattern among adolescent Nigerians attending secondary schools, they found prevalence of pre - hypertension among males and females were 14.3% and 20.1% respectively [15]. The finding in this study could be explained through analysis done that girls are more likely to have higher body fat percentage and more likely to be overweight and obesity when compared to boys.

Elevated BP was found to be high in those children studying in private schools than those children studying in public schools. These findings were anticipated given that those children studying in private schools came from families with higher social economic status than those studying in public schools. Wealthier families can afford electronic recreational games such as computer and video games. This has an impact to children's level of physical activity. Obesity and overweight showed significant association with elevated BP. This finding agrees with the study done by Tringler *et al*. [3] and Muhihi *et al*. [19] where they got similar findings. Our study identified several factors related to dietary and behavioral practices to be associated with elevated BP. Respondents who reported to usually eat fried food and drinking sugar soft drinks were more likely to have elevated BP than those who reported to not usually eat fried food nor drink sugar soft drinks. Additionally, not eating fruits was found to be significant associated with elevated BP among primary school children. This association has been demonstrated by another study conducted by Tringler *et al*. [3] who found that students with sedentary habits were almost 4 fold more likely to develop high blood pressure than their physically active counterparts.

The most important finding of this study is that there was a positive association between pocket money giving and elevated BP. Children who have been given money have had elevated BP than children not given money. They said, they bought sweets (peremende), cassava, fruit juices, groundnuts, ice cream, doughnut (andazi), potatoes, samosa, tea, baobab (ubuyu) and unrefined sugar (sukari guru). Generally, these bites are not good for health because they contain too much oils and sugar. In addition, television watching, video/computer gaming were thought to have an association with elevated BP, but results showed negative association. This could be explained by the fact that active video games may help children to raise their physical activity levels. Active video games played at moderate or vigorous intensity levels help them to be more active and expend more energy than adult. However, this is contrary to Tringler *et al*. [3] who reported that sedentary habits in children, television watching, video/computer gaming had strong association with elevated BP.

**Limitations:** 1) school children were given questionnaires to take home and parents helped them to fill in. This could have introduced bias and we might have missed some relevant information related to elevate BP since parents' education level was not checked. 2) Parents were unable to recall the birth weight of their children. 3) Presence of co - morbidities suggesting HTN was not checked.

## Conclusion

Findings showed that a significant proportion of primary school children in Mwanza region have elevated BP. Furthermore, results indicate that prevalence of elevated BP is higher among females than males, older children than young children, children eating fried food, studying in private schools, drinking sugar soft drinks. Furthermore, factors related to elevate BP among children that may be considered for future interventions in school children were identified.

**Recommendations:** 1) information to be communicated to schools where the study was conducted as well as to parents whose children had elevated BP. 2) In our settings evaluation of blood pressure in children and adolescents is not routine done, we emphasize the importance of blood pressure measuring among children. 3) We recommend using population specific reference ranges derived from particular study to rule out hypertension. 4) Parents need to be advised to set aside time for healthy meals, physical activity and limit giving children money. They should give their children food to take with them to school. 5) More research on this subject matter to further explore other factors for elevated BP that were not explored by this study such as genetic factors and birth weight.

### What is known about this topic

- Overweight and obesity were associated with increased risk of elevated BP;
- Age is associated with elevated BP whereby older children significantly have elevated BP than young children;
- Girls have higher BP than boys, other studies have reported similar pattern.

### What this study adds

- The most important finding of this study is that there was a positive association between pocket money giving and elevated BP since they bought unhealthy food at school;
- Television watching, video/computer gaming have no association with elevated BP, this could be explained by the fact that active video games may help children to raise their physical activity levels.

## References

[1] Urrutia-Rojas X, Egbuchunam CU, Bae S, Menchaca J, Bayona M, Rivers PA (2006). High blood pressure in school children: prevalence and risk factors. BMC Pediatr.

[2] Bassareo PP, Mercuro G (2014). Pediatric hypertension: An update on a burning problem. World J Cardiol.

[3] Tringler M, Rodriguez EM, Aguera D, Molina JD, Canziani GA, Diaz A (2012). High Blood Pressure, Overweight and Obesity Among Rural Scholars from the Vela Project. High Blood Press Cardiovasc Prev.

[4] World Health Organization (2000). Obesity: preventing and managing the global epidemic.

[5] Muthuri S, Wachira LJ, Leblanc A, Francis C, Sampson M, Onywera V (2014). Tremblay, Temporal trends and correlates of physical activity, sedentary behaviour, and physical fitness among school-aged children in sub-Saharan Africa: a systematic review. International journal of environmental research and public health.

[6] Zhu BQ, Parmley WW (1995). Hemodynamic and vascular effects of active and passive smoking. Am Heart J.

[7] Robinson RF, Batisky DL, Hayes JR, Nahata MC, Mahan JD (2005). Significance of heritability in primary and secondary pediatric hypertension. Am J Hypertens.

[8] Costanzi CB, Halpern R, Rech RR, de Araújo Bergmann ML, Alli LR, de Mattos AP (2009). Associated factors in high blood pressure among schoolchildren in a middle size city, southern Brazil. J Pediatr (Rio J).

[9] Maiti M, Bandyopadhyay L (2017). Variation in blood pressure among adolescent schoolchildren in an urban slum of Kolkata, West Bengal. Postgrad Med J.

[10] Amritanshu K, Kumar A, Pathak A, Garg N, Banerjee P (2015). Prevalence and risk factors associated with hypertension in children and adolescents. Pediatric Oncall.

[11] Chiolero A, Cachat F, Burnier M, Paccaud F, Bovet P (2007). Prevalence of hypertension in schoolchildren based on repeated measurements and association with overweight. J Hypertens.

[12] Maldonado J, Pereira T, Fernandes R, Santos R, Carvalho M (2011). An approach of hypertension prevalence in a sample of 5381 Portuguese children and adolescents. The AVELEIRA registry. “Hypertension in Children”. Blood pressure.

[13] Agyemang C, Redekop WK, Owusu-Dabo E, Bruijnzeels MA (2005). Blood pressure in rural, semi-urban and urban children in the Ashanti region of Ghana, West Africa. BMC Public Health.

[14] Noubiap JJ, Essouma M, Bigna JJ, Jingi AM, Aminde LN, Nansseu JR (2017). Prevalence of elevated blood pressure in children and adolescents in Africa: a systematic review and meta-analysis. The Lancet Public Health.

[15] Ujunwa FA, Ikefuna AN, Nwokocha AR, Chinawa JM (2013). Hypertension and prehypertension among adolescents in secondary schools in Enugu, South East Nigeria. Ital J Pediatr.

[16] Also U, Asani M, Ibrahim M (2016). Prevalence of elevated blood pressure among primary school children in Kano Metropolis, Nigeria. Nigerian Journal of Cardiology.

[17] Chillo P, Wakatare J, Janabi M, Matuj W, Greve G (2009). Low prevalence of cardiovascular risk factors among primary school children in Tanzania: an opportunity for primordial prevention?. Tanzania Medical Journal.

[18] Mushengezi B, Chillo P (2014). Association between body fat composition and blood pressure level among secondary school adolescents in Dar es Salaam, Tanzania. Pan Afr Med J.

[19] Muhihi AJ, Njelekela MA, Mpembeni RN, Muhihi BG, Anaeli A, Chillo O (2018). Elevated blood pressure among primary school children in Dar es salaam, Tanzania: prevalence and risk factors. BMC Pediatr.

[20] Mosha NR, Mahande M, Juma A, Mboya I, Peck R, Urassa M (2017). Prevalence, awareness and factors associated with hypertension in North West Tanzania. Glob Health Action.

[21] Mfinangai S, Kivuyo S, Ezekiel L, Ngadaya E, Mghamba J, Ramaiya K (2011). Public health concern and initiatives on the priority action towards non-communicable diseases in Tanzania. Tanzan J Health Res.

[22] Kavishe B, Biraro S, Baisley K, Vanobberghen F, Kapiga S, Munderi P (2015). High prevalence of hypertension and of risk factors for non-communicable diseases (NCDs): a population based cross-sectional survey of NCDS and HIV infection in Northwestern Tanzania and Southern Uganda. BMC Med.

[23] Chami N, Kabyemera R, Masoza T, Ambrose E, Kimaro F, Kayange N (2019). Prevalence and factors associated with renal dysfunction in children admitted to two hospitals in northwestern Tanzania. BMC Nephrol.

[24] Liz S (2005). New AHA recommendations for blood pressure measurement: American Heart Association Practice Guidelines. Am Fam Physician.

[25] Flynn JT, Kaelber DC, Baker-Smith CM, Blowey D, Carroll AE, Daniels SR (2017). Clinical practice guideline for screening and management of high blood pressure in children and adolescents. Pediatrics.

[26] Lurbe E, Cifkova R, Cruickshank JK, Dillon MJ, Ferreira I, Invitti C (2009). Management of high blood pressure in children and adolescents: recommendations of the European Society of Hypertension. J Hypertens.

[27] Yan W, Liu F, Li X, Wu L, Zhang Y, Cheng Y (2013). Blood pressure percentiles by age and height for non-overweight Chinese children and adolescents: analysis of the china health and nutrition surveys 1991-2009. BMC Pediatr.

[28] Krishna P, PrasannaKumar K, Desai N, Thennarasu K (2006). Blood pressure reference tables for children and adolescents of Karnataka. Indian pediatrics.

[29] WHO Multicentre Growth Reference Study Group (2006). Reliability of anthropometric measurements in the WHO Multicentre Growth Reference Study. Acta Paediatr Suppl.

[30] Lackland DT (2005). Racial disparities in hypertension. The Journal of Clinical Hypertension.

[31] Lackland DT (2014). Racial differences in hypertension: implications for high blood pressure management. The American journal of the medical sciences.

[32] Poor KM, Ducklow TB (2012). Benefit of BP measurement in pediatric ED patients. ISRN Nurs.

